# The Epidemiology of Low- and High-Energy Distal Radius Fracture in Middle-Aged and Elderly Men and Women in Southern Norway

**DOI:** 10.1371/journal.pone.0043367

**Published:** 2012-08-24

**Authors:** Andreas P. Diamantopoulos, Gudrun Rohde, Irene Johnsrud, Inger M. Skoie, Marc Hochberg, Glenn Haugeberg

**Affiliations:** 1 Department of Rheumatology, Hospital of Southern Norway, Kristiansand, Norway; 2 Faculty of Health and Sport, University of Agder, Kristiansand, Norway; 3 Departments of Medicine, Epidemiology and Preventive Medicine, University of Maryland School of Medicine, Baltimore, Maryland, United States of America; 4 Department of Neurosciences, INM, Norwegian University of Science and Technology, Trondheim, Norway; University of Southampton, United Kingdom

## Abstract

**Background:**

Distal radius is one of the most frequent sites for fractures in the elderly population. Despite this, there is a paucity of epidemiological data for distal radius fracture, in particular, distinguishing between high- and low-energy fractures. Our aim was to study the epidemiology of high- and low-energy distal radius fracture in middle-aged and elderly men and women in Southern Norway, and search for associates with high- or low-energy distal radius fracture in this population.

**Methodology/Principal Findings:**

Patients with distal radius fractures aged ≥50 years were identified from all four hospitals in Southern Norway between 2004 and 2005. Age-adjusted and age-specific incidence rates for men and women were calculated, and potential associates with high- and low-energy distal radius fracture were explored both in univariate and multivariate analyses. A total of 799 individuals (118 men and 681 women) aged ≥50 years with low-energy and 84 (48 men and 36 women) with high-energy distal radius fracture were identified. The overall age-adjusted incidence rate per 10,000 person-years was 18.9 for men (low energy, 12.8 vs. high-energy, 6.1) and 75.1 for women (low energy, 71.1 vs. high energy, 4.0). In multivariate model, younger age, male gender, summer season, and living in a rural area were independently associated with an increased risk of high-energy fracture.

**Conclusion:**

An approximately fourfold higher age-adjusted incidence rate for distal radius fracture was found among women, when compared with men. However, the proportion of patients with high-energy distal radius fracture was approximately fivefold higher in men than in women. Our data suggest that younger age, male gender, summer seasons, and living in rural areas are independent risk factors for increased risk of high-energy distal radius fracture.

## Introduction

The distal radius is one of the most frequent sites for fractures in elderly men and women [1]. Despite this there is a paucity of epidemiological data about distal radius fracture [2], [3], [4], [5], in particular distinguishing between low- and high-energy fractures [2]. Similar to the hip fracture, the incidence rates of distal radius fracture are reported to be higher in Scandinavia than in other European countries [6]. With regard to distal radius fracture, the highest incidence rates worldwide have been reported from urban areas in Western (Bergen) and Eastern (Oslo) Norway [2], [5]. Several factors associated with an increased risk of low-energy distal radius fracture have been identified, e.g. gender, vitamin D deficiency, seasonal variations, environmental conditions (ice and snow), medication (glucocorticosteroids), and osteoporosis [7], [8], [9], [10].

Our aim was to study the epidemiology of all distal radius fracture in middle-aged and elderly men and women in Southern Norway. Further, we also searched for factors associated with high-energy distal radius fracture in this population.

## Materials and Methods

The distal radius fracture patients were identified from the four regional hospitals (Kristiansand, Arendal, Flekkefjord, and Mandal) located in the two counties Vest-Agder and Aust-Agder in Southern Norway. Individuals aged ≥50 years residing in the two counties with a distal radius fracture between 1 January 2004 and 31 December 2005 were included. We excluded patients without residency in the two counties as well as those with distal radius fracture living in a small community in Vest-Agder County (Sirdal municipality) because some of the distal radius fracture patients living in this area theoretically may have been referred to the neighbor county hospital (Stavanger University Hospital, Rogaland County, West Norway).

The four hospitals are the only referral centers for orthopedic trauma in the two counties. For all individuals, we collected data on gender, date of birth, date of distal radius fracture, and place of residency. The electronic diagnosis registers at the hospitals were used to identify the distal radius fracture patients in the 2-year period, and were coded as S52.5 (fracture of lower end of radius), S52.6 (fracture of lower end of both radius and ulna), and S62.8 (fracture of other and unspecified parts of wrist and hand), according to the International Classification of Diseases 10th Revision (ICD-10). The identified patients' medical records and written X-ray reports were examined, and the diagnosis of distal radius fracture was confirmed before being included in the study. Low-energy fracture was defined as a result of falling from standing height or less, while high-energy fracture was defined as any other type of trauma (e.g. falling from height higher than standing height and motor vehicle accident). The medical records and written X-rays reports were initially reviewed by three rheumatologists (IMS, IJ, and AD) and if there was any doubt, AD and GH (senior rheumatologist) reviewed again the reports to reach a final conclusion.

To calculate the annual incidence of distal radius fracture in individuals aged ≥50 years over the 2-year period, we used the official population numbers of the two counties Vest-Agder and Aust-Agder, published online by Statistics Norway [11], excluding the population in Sirdal municipality.

Age-adjusted and age-specific fracture rates for both the genders were calculated. We also calculated gender-specific age-adjusted incidence rates for rural and urban areas. Urban areas were defined as municipals with >5000 citizens and >50% densely populated areas, a definition used by the official statistics bureau in Norway (SSB) [12]. To compare our age-adjusted incidence rates of distal radius fracture with those in previous reports, all incidence rates in these studies with available data were standardized to the mean population of the examined geographic area in 2004–2005 in Southern Norway [11].

Categorical variables were expressed as numbers or percentages and continuous variables were expressed as means with standard deviations (SD). For group comparison, we used the z-test. Distal radius fracture rates (number of fractures per 10,000 patient-years) were defined in 5-year intervals for the whole population of patients and for each gender separately. The incidence in different age groups was calculated as the number of distal radius fractures divided by the mean population. The 95% confidence intervals (CI) for the incidence rates and seasonal prevalence of distal radius fractures were calculated using the equation for binominal distribution [13]. The association between high- and low-energy distal radius fractures as dependent variable and age, gender, seasons, and urban/rural areas as independent variables was tested both in univariate and multivariate analysis. The statistical analyses were performed with the StatCalc 2.6 program (AcaStat Software, Leesburg, VA) or SPSS (SPSS Inc. Chicago Ill.), and values of *p*<0.05 were considered to be significant.

The study was approved by the Regional ethics committee.

## Results

In the 2-year period, among residents living in the recruitment area aged ≥50 years, a total of 883 (166 men and 717 women) individuals were identified with a distal radius fracture. Among them, 799 (118 men and 681 women) had a low-energy and 84 (48 men and 36 women) a high-energy distal radius fracture. Among the distal radius fracture patients 14 patients also had a concomitant hip fracture.

The proportion of patients with high-energy distal radius fracture was significantly higher in men than in women (28.9% vs. 5.9%, *p*<0.001). The mean age for all distal radius fracture patients was 69.5 years (65.3 years for men, 70.5 years for women, *p*<0.001), that for low-energy fracture patients was 70.1 years (66.8 years for men, 70.7 years for women, *p* = 0.001), and that for high-energy fracture patients was 63.7 years (61.7 years for men, 66.3 years for women, *p* = 0.032).

### Age-adjusted and age-specific distal radius fracture incidence rates in men and women

For all distal radius fractures, the mean (95% CI) age-adjusted incidence rate per 10,000 person-years was 18.9 (15.9–21.9) for men and 75.1 (69.1–81.1) for women. The corresponding figures for low-and high-energy fracture in men were 12.8 (9.8–15.8) and 6.1 (4.1–8.1), and for women were 71.1 (66.1–76.1) and 4.0 (3.0–5.0), respectively.

**Figure 1:**
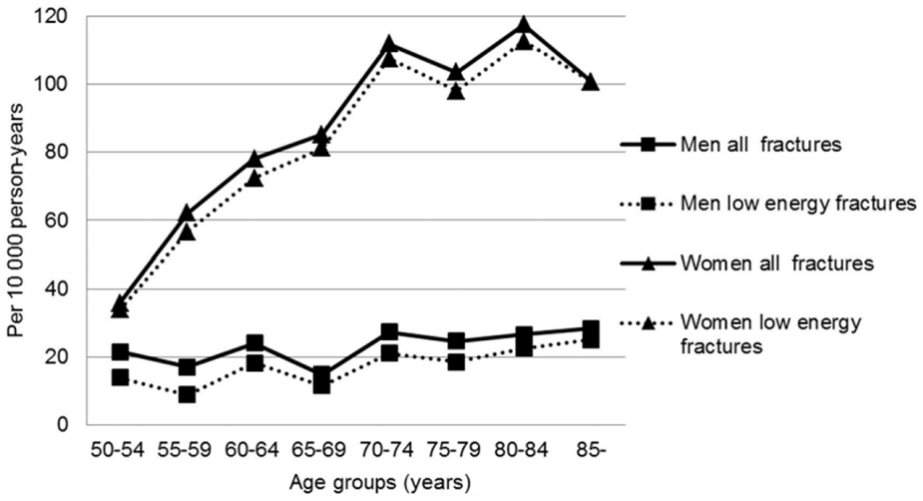
Age-specific incidence rates for all and low-energy distal radius fracture in men and women in Southern Norway (2004–2005).

As shown in Table 1 and Figure 1, the age-specific incidence rates of distal radius fracture in men increased slightly with age. On the other hand, for women, the age-specific incidence rates increased linearly from the age of 50 years, stabilized at the age of 70–84 years, and slightly decreased at the age of ≥85 years.

**Table 1:** Number and average annual incidence of distal radius fracture per 10,000 person-years with 95% confidence intervals (CI) for different age groups in men and women in Southern Norway (January 2004–December 2005).

	Men					Women				
	Person-years	# All fractures	# Low-energy fractures	Annual incidence All fractures	Annual incidence Low-energy fractures	Person-years	# All fractures	# Low-energy fractures	Annual incidence All fractures	Annual incidence Low-energy fractures
50–54 years	17,169	37	24	21.5 (14–28)	13.9 (8–20)	16,778	60	57	35.8 (26–44)	33.9 (25–43)
55–59 years	16,952	29	15	17.1 (11–23)	8.8 (4–14)	16,413	102	93	62.1 (50–74)	56.6 (45–69)
60–64 years	12,041	29	22	24.1 (15–33)	18.2 (10–26)	12,562	98	91	78.0 (63–93)	72.4 (57–87)
65–69 years	9466	14	11	14.8 (6–22)	11.6 (5–19)	9972	85	81	85.2 (67–103)	81.2 (63–99)
70–74 years	8048	22	17	27.3 (16–38)	21.1 (11–31)	9209	103	99	111.8 (90–132)	107.5 (86–128)
75–79 years	6486	16	13	24.6 (12–36)	18.5 (8–28)	9088	94	89	103.4 (82–124)	97.9 (78–118)
80–84 years	4882	13	11	26.6 (12–40)	22.5 (9–35)	8264	97	93	117.4 (94–140)	112.5 (89–135)
85+ years	3177	9	8	28.3 (10–46)	25.1 (8–42)	7 452	75	75	100.6 (77–123)	100.6 (77–123)

Furthermore, a statistically significant difference in age-specific incidence rates between the two genders was observed for all age groups. However, no statistically significant difference was noted between the age-specific incidence rates of all and low-energy distal radius fractures (Figure 1).

### Rural and Urban areas

The age-adjusted incidence rates for all distal radius fractures were statistically significantly higher for men living in urban areas, when compared with those living in rural areas [23.8 (19.8–27.8) vs.12.8 (7.8–17.8), *p* = 0.005]. The same trend was observed among women, but the difference between urban and rural areas was not statistically significant [82.6 (75.6–89.6) vs. 69.0 (58.0–80.0), *p* = 0.06]. With regard to low-energy distal radius fracture, the higher incidence rates in urban areas, when compared with those in rural areas, were statistically significant both for men [17.1 (14.1–20.1) vs. 8.5 (4.5–12.5), *p*<0.01)] and women [79.7 (72.7–86.7) vs. (62.5 (51.5–73.5), *p* = 0.013)].

### Seasonal variation

The number of all distal radius fractures and low-energy distal radius fractures was higher in winter (*p*<0.001), when compared with each of the other seasons (Table 2). However, no statistically significant differences in the fracture rates were observed between spring, summer, and autumn for all distal radius fractures. On the other hand, a statistically significant higher rate of low-energy distal radius fracture was noted between spring and summer, and spring and autumn (*p*<0.01).

**Table 2:** Prevalence of distal radius fractures in Southern Norway in the 2-year period 2004–2005 stratified according to seasons. Data presented as numbers and percentage with 95% confidence interval (CI) for all and low-energy fractures.

Seasons	Number of fractures		% of fractures (95% CI)	
	All	Low energy	All	Low energy
Winter (December–February)	311	291	35.2 (32.0–38.3)	36.4 (36.0–39.7)
Spring (March–May)	213	194	24.1 (21.2–26.9)	24.3 (21.3–27.2)
Summer (June–August)	179	154	20.3 (17.6–22.9)	19.3 (16.5–22.0)
Autumn (September–November)	180	160	20.4 (17.7–23.0)	20.0 (17.2–22.7)

### Associates with high-energy distal radius fractures

As shown in Table 3, younger age, male gender, and summer season were significantly associated with an increased risk of high-energy distal radius fracture both in univariate and multivariate analyses. On the other hand, living in rural area was found to be marginally significantly associated in the univariate analysis and significantly associated in the multivariate analysis with an increased risk of high-energy distal radius fracture.

**Table 3:** Associates with high-energy distal radius fracture in individuals aged ≥50 years tested both in univariate and multivariate logistic regression models.

	Variables tested in a univariate model		Variables tested in a multivariate model	
	OR (95% CI)	*p-*value	OR (95% CI)	*p-*value
Age groups (years)				
50.0–59.9 (ref. group)				
60.0–69.9	0.48 (0.27–0.84)	*0.01*	0.57 (0.31–1.04)	0.07
70.0–79.9	0.40 (0.22–0.72)	*0.01*	0.50 (0.26–0.94)	*0.03*
80.0	0.18 (0.08–0.41)	<0.001	0.24 (0.10–0.57)	*0.01*
Women (ref. group)/men	7.69 (4.80–12.36)	*<0.01*	7.01 (4.27–11.51)	*<0.01*
Urban (ref. group)/rural residence	1.63 (0.97–2.73)	0.06	2.08 (1.17–3.70)	*0.01*
Seasons				
Winter (ref. group)				
Spring	1.42 (0.74–2.74)	0.29	1.50 (0.75–3.01)	0.25
Summer	2.36 (1.27–4.39)	*0.01*	2.38 (1.22–4.66)	*0.01*
Autumn	1.82 (0.95–3.48)	0.07	1.99 (0.94–3.77)	0.07

OR: Odds ratio. CI: Confidence intervals. Ref.: reference.

## Discussion

In line with the previous reports, we found that age-adjusted incidence rates for all distal radius fractures for individuals aged ≥50 years, living in Southern Norway, were higher in women than in men. In our study, we found an approximately 4∶1 female-male ratio, whereas other Norwegian studies have reported a 5∶1 female-male ratio [2], [5]. No statistically significant difference in age-adjusted incidence rates for high-energy fracture was found between men and women. When high-energy fracture patients were excluded, the age-adjusted incidence rates per 10,000 person-years for distal radius fracture decreased from 18.9 to 12.8 in men and from 75.1 to 71.1 in women. Our data thus give an indication of the contribution of high-energy distal radius fracture on the total incidence rate of distal radius fracture in middle-aged and elderly men and women. In our study, high-energy distal radius fracture was associated with male gender, younger age, summer season, and living in rural areas. The incidence rates of low-energy distal radius fracture were higher in women as well as in winter, when compared with those in the other seasons, and were higher in urban than in rural areas.

We observed a steady increase in distal radius fracture in women until the age of 70 years. After this age, the incidence rates stabilized and thereafter decreased slightly in the age group of ≥85 years. In men, the incidence rates were more stable across all age groups. These results are in agreement with those reported in previous published studies [5], [14]. However, in our study, the risk of having a high-energy distal radius fracture was decreasing with increasing age. This is possibly due to decreased recreational activities in older adults and thus reduced risk for high-energy trauma. The increase in low-energy fracture with age may be explained by the increasing number of patients with osteoporosis, especially women [8]. The higher incidence rates of distal radius fracture in summer in younger men living in rural areas may be explained by the higher degree of activity during this period, and because people in rural areas are physically more active than those living in urban areas and thus more exposed to trauma [15], [16], [17]. The increased risk of high-energy distal radius fracture in men than in women may be explained by the fact that men do more physical hard work and more hazardous labor activity. In the study by Hove et al., it was reported that distal radius fracture during work and sports activity is more often in men than in women [2].

In our study carried out in Southern Norway, as shown in Table 4, we found a significantly lower age-adjusted distal radius fracture incidence rates among women, when compared with the previous reports of 1999 and 1988 from Oslo city, the capital of Norway [5], and Bergen, the second largest city in Norway [2], respectively. Our data are more in line with the previous reports from Denmark, Sweden, and Finland [3], [4], [18], but higher than those reported from, e.g., the UK and Taiwan (Table 4). However, for men, we did not find any significant differences between our reported incidence rates from Southern Norway and those from Oslo and Bergen [2], [5]. For men, as shown in Table 4, the 95% CI for the incidence rates was found to be overlapping in all comparable studies, except for the studies from Denmark and UK, which reported a significant lower incidence rate than that found in our study.

**Table 4:** Age-adjusted distal radius fracture rates for Norway, Scandinavia, and other selected countries among men and women older than 50 years.

Region, Country, and time period [reference]	Study population		Number of fractures		Age adjusted incidence rates (95% CI)	
	Person-years men	Person-years women	Men	Women	Men	Women
*Southern Norway 2004–2005 (present study)*	*78,221*	*89,740*	*169*	*714*	*19 (16–22)*	*75 (69–81)*
Bergen, Norway 1988 [2]	75,813	83,124	128	481	18 (15–21)	109 (102–116)
Oslo, Norway 1998–1999 [5]	64,871	85,986	165	944	25 (21–29)	106 (99–113)
Uppsala, Sweden 1989–1990 [4]	34,493	40,537	102	523	23 (18–28)	94 (85–103)
Fredriksborg, Denmark 1981 [3]	111,000	114,000	99	394	12 (10–14)	87 (82–92)
Dorset, UK 1997* [25]	61,102	72,962	98	896	12 (9–15)	66 (60–72)
Oulu, Finland 2008 [18]	18,366	23,093	41	164	15 (9–21)	73 (64–86)
Taiwan, 2007 [26]	Whole Taiwan population	Whole Taiwan population	161	558	13**	31**

The rates are per 10,000 patient-years.

CI: Confidence intervals.

* The Dorset, UK, incidence rates are calculated for men and women older than 55 years.

** Not possible to calculate the CI.

The lower incidence rates of distal radius fracture in our study could reflect a falling tendency of incidence rates over the last decades. In a study carried out in the Northeastern part of Sweden in 2001, it has been reported that the age-adjusted incidence rate of distal radius fracture is approximately 30% lower than that reported in Uppsala in Southern Sweden at the end of the 1980s [4], [19]. In a study carried out in Oslo, the overall incidence rate for distal radius fracture in women over a period of approximately 20 years, from 1979 to 1998, was found to be stable [5]. However, in the Oslo study, all the forearm, elbow, and distal radius fractures were included, and this could partially explain the higher incidence rates than those reported in our study [5]. With regard to hip fracture, we had recently reported a significant lower age-adjusted incidence rate in Southern Norway, when compared with that observed in Oslo [20]. An explanation for this could be the differences in the population demographics between Southern Norway and Oslo. The number of women living alone in the Oslo area is higher in all age groups than the rest of the country [21]. This may contribute to the higher distal radius fracture risk reported in Oslo, because living alone has been reported to be associated with an increased risk of distal radius fracture, independent of other known risk factors such as age, weight, and osteoporosis [9]. It may also be possible that the difference in the reported incidence rates of distal radius fracture between the study carried out in Oslo and our study reflects the degree of urbanization of the geographic area studied.

Differences in bone density between the geographic regions may also explain the differences in the incidence of distal radius fracture. In a study comparing hip bone mineral density (BMD) between the population of Tromsø city located in Northern Norway and Bergen city located in Western Norway, significant differences were found in BMD, with women older than 60 years and men of any age living in Tromsø having higher BMD than those living in Bergen [22]. Thus, in general, the large burden of osteoporosis in the Scandinavian countries may partly explain the high incidence rates of distal radius fracture reported in these countries [23].

In our study, we found a statistically significant lower age-adjusted incidence rate for distal radius fracture in men living in rural areas, when compared with those living in urban areas, and a marginally significant lower rate in women. However, when only the low-energy fractures were included, this difference was statistically significant for both the genders. This may be explained by the fact that people living in rural areas are more physically active and thus have a better bone health than those living in urban areas [15], [16], [17]. In a recently published Norwegian prospective study examining the risk of distal radius fracture in the population in the Central and Northern part of Norway, women living in urban areas were found to be at a higher risk of having a distal radius fracture than those living in rural areas. However, when adjusted for bone density, no significant difference was observed in the distal radius fracture risk between women living in urban and rural areas [15]. The importance of bone density as a risk factor for fragility distal radius fracture both in men and women has also been reported in other studies [8], [9], [10], [24]. In addition, greater rates of low-energy distal radius fractures in urban areas could reflect different degrees of comorbidity and, therefore, an increased risk to fall.

In our study, the incidence of low-energy distal radius fracture was higher in winter than the other seasons, similar to that reported in other studies [2], [18]. The higher prevalence of distal radius fracture in winter months may be explained by an increased risk of falling caused by slippery condition on snow and ice [2], [9], [18].

The limitations of our study are as follows. The retrospective data collection method from the four hospitals using the ICD diagnosis coding system may not have identified all distal radius fracture patients due to false ICD-10 coding. Further, patients residing in the geographic area with a distal radius fracture outside our catchment area would not have been identified and included. Both these limitations may have underestimated the incidence of distal radius fracture. Ideally, we should have reviewed the diagnostic X-rays confirming the presence of a distal radius fracture in the study population. Thus, we cannot exclude that some of the patients treated for distal radius fracture, in fact did not have a distal radius fracture.

In summary, in our study, we found a significant lower incidence rate of distal radius fracture among women, but not among men, when compared with the previous reports from Norway. Our study also demonstrated that male gender, living in a rural area, and summer season were all independently associated with an increased risk of having a high-energy fracture, whereas higher age was found to be associated with a decreased risk of high-energy distal radius fracture. Our data contributes to an increased understanding of the epidemiology of distal radius fracture in middle-aged and elderly men and women.
